# Performance of *HLA* allele prediction methods in African Americans for class II genes *HLA-DRB1*, −*DQB1*, and *–DPB1*

**DOI:** 10.1186/1471-2156-15-72

**Published:** 2014-06-16

**Authors:** Albert M Levin, Indra Adrianto, Indrani Datta, Michael C Iannuzzi, Sheri Trudeau, Paul McKeigue, Courtney G Montgomery, Benjamin A Rybicki

**Affiliations:** 1 Department of Public Health Sciences, Henry Ford Health System, 1 Ford Place, 3E, 48202 Detroit, MI, USA; 2 Center for Bioinformatics, Henry Ford Health System, Detroit, MI, USA; 3 Arthritis and Clinical Immunology Research Program, Oklahoma Medical Research Foundation, Oklahoma City, OK, USA; 4 Department of Medicine, Upstate Medical University, Syracuse, NY, USA; 5 Public Health Sciences Section, University of Edinburgh Medical School, Edinburgh, Scotland

**Keywords:** *HLA*, African American, Single nucleotide polymorphisms, Imputation, Admixture

## Abstract

**Background:**

The expense of human leukocyte antigen (*HLA*) allele genotyping has motivated the development of imputation methods that use dense single nucleotide polymorphism (SNP) genotype data and the region’s haplotype structure, but the performance of these methods in admixed populations (such as African Americans) has not been adequately evaluated. We compared genotype-based—derived from both genome-wide genotyping and targeted sequencing—imputation results to existing allele data for *HLA–DRB1*, −*DQB1*, and *–DPB1*.

**Results:**

In European Americans, the newly-developed HLA Genotype Imputation with Attribute Bagging (HIBAG) method outperformed HLA*IMP:02. In African Americans, HLA*IMP:02 performed marginally better than HIBAG pre-built models, but HIBAG models constructed using a portion of our African American sample with both SNP genotyping and four-digit *HLA* class II allele typing had consistently higher accuracy than HLA*IMP:02. However, HIBAG was significantly less accurate in individuals heterozygous for local ancestry (p ≤0.04). Accuracy improved in models with equal numbers of African and European chromosomes. Variants added by targeted sequencing and SNP imputation further improved both imputation accuracy and the proportion of high quality calls.

**Conclusion:**

Combining the HIBAG approach with local ancestry and dense variant data can produce highly-accurate *HLA* class II allele imputation in African Americans.

## Background

The human leukocyte antigen (*HLA*) region resides within the major histocompatibility complex (*MHC*) on chromosome 6p21.31 and contains multiple genes encoding highly variable antigen-presenting proteins that play a key role in immunity [1]. Among these, the class I genes *HLA–A*, −*B*, and *–C*, and the class II genes *HLA–DRB1*, −*DQB1*, −*DQA1*, and *–DPB1* are the most frequently studied, Decades of *HLA* research have revealed that genetic variation in these genes play important roles in disease susceptibility and pharmacogenetic interactions that influence the efficacy of certain drugs.

The nomenclature developed to catalogue the allelic variation in *HLA* genes has evolved over time to incorporate a growing number alleles identified in each gene. In 1987, a four-digit code (e.g. HLA-DRB1*0401) was first employed to catalogue alleles that differed in protein sequence [2]. The first two digits correspond to the protein serotypes distinguishable by serological reagents used before polymerase-chain reaction-based methods were available [3]. Coupled with the first two digits, the second two specify a unique amino acid sequence or, equivalently, haplotypes of non-synonymous (protein-altering) polymorphisms within each gene. Subsequently, a colon was introduced into the notation (e.g. HLA-DRB1*04:01) to separate the digits into fields (*e.g.* first field corresponding to serotype and the second to differences in haplotypes of non-synonomous polymorphisms) to accommodate an ever-increasing number of alleles [4]. While additional fields have been added to delineate alleles that differ in genetic variation that does not alter the protein amino acid sequence, the functional two-field alleles remain the primary focus in basic research and clinical applications.

In particular, prior to the advent of genome-wide genotyping and sequencing technologies, typing of the two-field alleles led to breakthroughs in our understanding of the role of *HLA* genes in the genetic architecture of multiple immune-mediated diseases [5], which have been replicated by genome-wide association studies (GWAS). However, few GWAS have been followed up by direct *HLA* allele typing to dissect the potentially multiple causal variants driving the observed associations at single nucleotide polymorphisms (SNPs), partially due to the high cost of genotyping the *HLA* alleles via sequence-specific oligonucleotide primers in GWAS of thousands of individuals.

As a result, imputation of *HLA* alleles using large reference panels—such as those assembled by the International HapMap consortium or the 1000 Genomes (1KG) project—has grown more common. While initial studies have shown that one or more SNPs may be used to “tag” common *HLA* alleles (allele frequency ≥0.05) within ancestral population groups [6], many of the *HLA* alleles are rare (allele frequency <0.05) in a given population and may not be reliably tagged by sets of two or three SNPs. Methods that address this challenge using dense SNP genotyping and known linkage disequilibrium and haplotype structure [7-13] have led to breakthroughs in identifying causal variants within *HLA*, including recent successes in rheumatoid arthritis [14] and multiple sclerosis [15]. While these studies demonstrated the power and efficiency of *HLA* imputation, use of these methods has generally been confined to samples of primarily European ancestry.

The extension of imputation methods to admixed populations is critical for mapping *HLA*-dependent diseases that differ in incidence between ancestral populations. The recently-developed HLA Genotype Imputation with Attribute Bagging (HIBAG) [16] and HLA*IMP:02 [13] methods are the first imputation methods to address *HLA* allele imputation in admixed populations, but these methods are new and include a limited number of admixed individuals in the published models. These methods also differ from one another, with HIBAG employing multiple expectation-maximization-based classifiers to estimate the likelihood of *HLA* alleles and HLA*IMP:02 using a haplotype graph-based approach. They are similar in that they allow researchers with GWAS genotyping but without *HLA* allele data from appropriate reference samples an option to impute the alleles in their own subjects. In the current study, we used genome-wide genotyping [17] and *HLA* allele data [18] from our previous studies of sarcoidosis to compare the imputation accuracy of these methods in both African American and European American individuals. We also investigated whether HIBAG models using these data improved upon existing model predictions for African Americans and evaluated the impact of local ancestry information on imputation accuracy in admixed subjects [19,20]. Finally, we determined the influence of increasing the SNP density through adding variation from SNP imputation and targeted sequencing on the imputation accuracy of *HLA* alleles.

## Results

Our sample comprising 2,727 African Americans (1,271 cases, 1,456 controls) and 2,726 European Americans (442 cases, 2,284 controls) has been described previously [17,21]. The African American samples were assembled from the following studies: 1) a case–control etiologic study of sarcoidosis (ACCESS) [22]; 2) a multi-site affected-sibling sarcoidosis linkage study [23]; 3) a nuclear family-based sample ascertained through a single affected individual within the Henry Ford Health System in Detroit, Michigan [24]; and 4) healthy controls from the Oklahoma Medical Research Foundation’s Lupus Family Registry and Repository [25]. European American sarcoidosis cases were derived from both the ACCESS and Henry Ford samples. Low- to intermediate-resolution *HL*A genotype data were available for a subset of subjects from the ACCESS study [22]: 325 African Americans (156 cases, 169 controls) and 480 European Americans (239 cases, 241 controls).

The published HIBAG models were applied to the sample of ACCESS European Americans (n = 480) with available *HLA*-typing and genome-wide genotyping to validate the one- and two-field allele classification accuracy. The overall imputation accuracy results for the *HLA–DRB1*, −*DQB1*, and *–DPB1* in European Americans are presented in Table 1; the allele-specific model performance measures (imputation accuracy, sensitivity, specificity, positive predictive value, and negative predictive value) are presented in Additional file 1. Imputation accuracy was high (>90%) at both the one- and two-field resolution across all three genes. Removal of subjects with posterior predicted genotype probabilities ≤0.5—a threshold calibrated by the developers of HIBAG—reduced the sample size (8.3% reduction for *–DRB1*, 0.8% for *–DQB1*, and 2.3% for *–DPB1*) but resulted in slightly improved classification rates. Compared to HLA*IMP:02, HIBAG had higher imputation accuracy at both one- and two-field resolution (Table 1) for all three genes, with the exception *HLA-DQB1* at two-field resolution, where the two methods produced comparable results. The largest difference was observed for *HLA–DPB1*, with 10.2% and 10.6% higher accuracy rates at the one- and two-field resolutions, respectively.

**Table 1:** **Comparison of *HLA *** **allele imputation accuracy in ACCESS European Americans for *HLA *** **class II genes *HLA-DRB1*, − *DQB1 *, and *−DPB1 *** between HLA*IMP:02* and HIBAG^**†**^

		HLA*IMP:02*		**HIBAG†**			
		**All**		**All**	**PP > 0.5**		
**Gene**	**Allele field**	**N**	**Accuracy**	**Accuracy**	**N**	**Call rate**	**Accuracy**
*DRB1*	One	480	98.5	98.9	440	91.7	99.8
	Two	480	90.4	92.4	440	91.7	94.4
*DQB1*	One	480	99.5	99.7	476	99.2	99.7
	Two	480	97.1	97.0	476	99.2	97.2
*DPB1*‡	One	480	83.0	93.2	469	97.7	93.6
	Two	480	82.1	92.7	469	97.7	93.2

Abbreviations: PP denotes, posterior prediction probability; N, count; Accuracy, imputation accuracy.

*HLA*IMP:02 is described in Dilthey et al. 2013.

†HIBAG European ancestry model (published online 8/10/2012).

‡One- and two-field estimates will be very similar for *HLA-DPB1* as the first field uniquely identifies the two-field alleles, with the exceptions of *02:01 and *0202, and *04:01 and *04:02.

In comparison to European Americans, classification accuracies were lower for African Americans (Table 2), and HIBAG published models were overall less accurate than HLA*IMP:02. Using the published HIBAG African ancestry models, imputation accuracy rates ranged from 69%–96% in the absence of a posterior predictive probability threshold (Table 2); the allele-specific performance measures are presented in Additional file 2. When the 0.5 threshold was applied, two-field resolution accuracy increased 5.3–19.8%. Compared to European Americans, the call rates for African American subjects that exceeded this threshold was substantially lower (47.7–65.8%). In contrast, HLA*IMP:02 African American call rates at the same threshold were higher (minimum value of 82.5%).

**Table 2:** ***HLA *** **allele imputation accuracy in ACCESS African Americans for *HLA *** **class II genes *HLA-DRB1*, − *DQB1 *, and *–DPB1***

			**Testing set**				
			**All**		**PP > 0.5**		
**Method**	**Gene**	**Allele field**	**N**	**Accuracy**	**N**	**Call rate**	**Accuracy**
HLA*IMP:02*	*DRB1*	One	325	93.2	268	82.5	94.8
		Two	325	83.8	268	82.5	87.3
	*DQB1*	One	325	96.3	297	91.4	97.3
		Two	325	93.2	297	91.4	94.6
	*DPB1*§	One	—	—	—	—	—
		Two	—	—	—	—	—
HIBAG†	*DRB1*	One	325	89.5	135	41.5	98.1
		Two	325	78.8	135	41.5	94.4
	*DQB1*	One	325	96.6	170	52.3	98.8
		Two	325	87.1	170	52.3	92.4
	*DPB1*§	One	325	69.5	111	34.2	89.2
		Two	325	69.4	111	34.2	89.2
HIBAG ACCESS‡	*DRB1*	One	164	95.4	113	68.9	99.1
		Two	164	89.6	113	68.9	96.0
	*DQB1*	One	188	95.4	166	88.3	99.7
		Two	188	97.6	166	88.3	99.1
	*DPB1*§	One	250	87.4	147	58.8	98.3
		Two	250	86.4	147	58.8	98.0
BEAGLE ACCESS‡	*DRB1*	One	164	78.7	—	—	—
		Two	164	72.3	—	—	—
	*DQB1*	One	188	96.0	—	—	—
		Two	188	97.9	—	—	—
	*DPB1*§	One	250	65.2	—	—	—
		Two	250	64.4	—	—	—

Abbreviations: PP denotes, posterior prediction probability; N, count; Accuracy, imputation accuracy.

*HLA*IMP:02 is described in Dilthey et al. 2013. In this version of the software, reference data for *HLA-DPB1* was not available for the African ancestry reference panel.

†HIBAG African ancestry model (published online 8/10/2012).

‡The models trained on the subset of ACCESS African Americans with training samples selected at random (from a total of 325) to match the number of subjects used to construct the published HIBAG African ancestry models: *HLA-DRB1* (n = 161), *HLA-DQB1* (n = 137), and *HLA-DPB1* (n = 75).

§One- and two-field estimates will be very similar for *HLA-DPB1* as the first field uniquely identifies the two-field alleles, with the exceptions of *02:01 and *0202, and *04:01 and *04:02.

**Figure 1:**
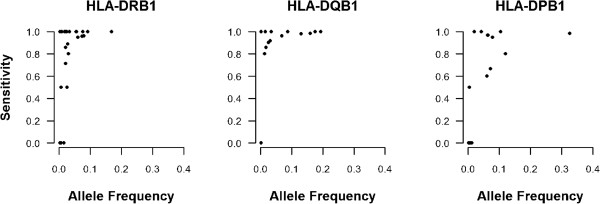
**African American allele prediction sensitivity for two-field *HLA *class II genes by allele frequency using the ACCESS HIBAG models.** Sensitivity is equal to the probability that the predicted allele matches the actual genotyped allele prediction (i.e. true positive/(true positive + false negative)).

Next, we constructed HIBAG models using the ACCESS African American sample as reference to analyze the imputation accuracy and quality of gene-specific prediction models (Table 2); the corresponding allele-specific performance measures are presented in Additional file 3. Samples not used for training were used to estimate the imputation accuracy of models (*i.e.* the test set). These models performed well (accuracy >86%) with 10.8%–21.8% higher imputation accuracy than published HIBAG African ancestry and HLA*IMP:02 models at two-field resolution; the ACCESS HIBAG models outperformed BEAGLE at *HLA–DRB1* and *–DPB1*, with comparable accuracy achieved for *HLA–DQB1*. Figure 1 displays the plots of ACCESS HIBAG allele sensitivity (i.e. proportion of a particular allele accurately predicted) by allele frequency for each of the three genes. For alleles with a frequency ≥ 1%, the median (interquartile range) of sensitivity were 98.1% (86.5%–100.0%), 98.3% (91.7%–100.0%), and 96.8% (73.3%–99.1%) for *HLA–DRB1*, −*DQB1*, and *–DPB1*, respectively. Also, A difference in the quality of the predictions was found, with 68.9% (−*DRB1*), 88.3% (−*DQB1*), and 58.8% (−*DPB1*) exceeding the posterior probability threshold of 0.5. Above this threshold, two-field resolution imputation accuracies were ≥ 96% across all genes.

To determine the relationship between posterior probability and imputation accuracy in the African American sample, we analyzed two-field accuracy estimates by HIBAG posterior probability thresholds (Table 3). For all three genes, imputation accuracy estimates exceeded 90% at a posterior probability threshold >30%, suggesting that this threshold is associated with high imputation accuracy levels in African Americans.

**Table 3:** HIBAG model* two-field *HLA * **allele imputation accuracy in the ACCESS African American gene-specific training sets by posterior probability threshold of the most likely genotype call**

	***DRB1***		***DQB1***		***DPB1***	
**Posterior probability range**	**N**	**Accuracy**	**N**	**Accuracy**	**N**	**Accuracy**
>0.10	163	89.8	188	97.6	249	86.3
>0.20	160	90.3	187	97.6	239	87.9
>0.30	149	92.2	184	97.8	213	90.6
>0.40	136	94.4	175	98.9	174	95.4
>0.50	113	95.8	166	99.1	147	98.0
>0.60	93	97.3	156	99.4	121	97.9
>0.70	70	98.6	142	99.3	87	97.1
>0.80	53	98.1	116	99.1	38	96.1
>0.90	28	96.4	74	99.3	13	96.2

Abbreviations: N denotes, count; Accuracy, imputation accuracy.

*The models trained on the subset of ACCESS African Americans with training samples selected at random (from a total of 325) to match the number of subjects used to construct the published HIBAG African ancestry models: *HLA-DRB1* (n = 161), *HLA-DQB1* (n = 137), and *HLA-DPB1* (n = 75).

The full sample of 325 African American subjects from ACCESS with both *HLA*-typing and genome-wide genotyping was also used for model training. Applying these models to the remaining 2,402 African Americans with genome-wide genotyping but without *HLA* typing, the proportion of samples with posterior prediction probabilities > 0.5 increased to 85.7% (−*DRB1*), 93.8% (−*DQB1*), and 90.3% (−*DPB1*), approaching the results seen in European Americans (Table 1).

Variable local ancestry may also impact *HLA* imputation accuracy in African Americans. Table 4 displays the ACCESS HIBAG imputation accuracy estimates by local ancestry status at each gene. Fisher’s exact tests indicate that differences in accuracy by local ancestry were evident at each of the genes at two-field resolution (p-values ≤ 0.04); accuracy was consistently 5.2–14.2% lower for heterozygous individuals (those with one African-derived DNA segment and one European-derived segment) compared to those homozygous for the West African ancestral haplotype. To determine whether these differences could be reduced, HIBAG models were trained on a mixed sample of 150 ACCESS European Americans (*i.e.* 300 European haplotypes) and 150 ACCESS African American with two West African alleles (*i.e.* 300 West African haplotypes) and tested on the remaining 175 ACCESS African Americans not included in the training sample (Table 5); the corresponding allele-specific performance measures for this test set are presented in Additional file 4. Using these mixed-ethnicity models, there were no longer statistically significant differences in two-field classification accuracy by local ancestry status (p > 0.1) at any of the three genes.

**Table 4:** HIBAG model* four-digit HLA allele prediction accuracy in ACCESS African Americans differs by local ancestry at HLA class II genes

**Gene**	**West African ancestral alleles†**	**N**	**Accuracy**	**P‡**
DRB1	2	240	92.9	0.005
	1	78	80.8	
	0	10	80.0	
DQB1	2	292	98.6	0.040
	1	76	93.4	
	0	8	100.0	
DPB1	2	376	89.6	8.8*10^-4^
	1	110	75.5	
	0	14	85.7	

Abbreviations: N=count; ACC=classification accuracy; P=p-value from a Fishers exact test.

*The models trained on the subset of ACCESS African Americans with training samples selected at random (from a total of 325) to match the number of subjects used to construct the published HIBAG African ancestry models: HLA-DRB1 (n=161), HLA-DQB1 (n=137), and HLA-DPB1 (n=75).

†Number of West African ancestral alleles at each gene estimated by the Local Ancestry in Admixed Populations (LAMP) method.

‡Test for difference in imputation accuracy proportions across individuals with 0, 1, or 2 West African Alleles at locus in question.

**Table 5:** **Local ancestry differences in two-field *HLA *** allele imputation accuracy resolved by HIBAG models containing equal numbers* of ancestral West African and European chromosomes

**Gene**	**West African ancestral alleles†**	**N**	**Accuracy**	**P‡**
*DRB1*	2	91	88.5	0.12
	1	75	81.3	
	0	9	94.4	
	Overall	175	85.7	
*DQB1*	2	91	95.1	1.00
	1	75	95.3	
	0	9	100.0	
	Overall	175	95.4	
*DPB1*	2	91	89.0	0.92
	1	75	89.6	
	0	9	94.4	
	Overall	175	90.6	

Abbreviations: N denotes, count; Accuracy, imputation accuracy; P, p-value from a Fishers exact test.

*150 individuals (300 chromosomes) from African American with two ancestral West African alleles at each gene as estimated by LAMP and 150 individuals from the ACCESS European American sample (i.e. 300 European chromosomes). The test set for these models included the remaining 175 African Americans who were not a part of the 150 used to build the model.

†Number of West African ancestral alleles at each gene estimated by the Local Ancestry in Admixed Populations (LAMP) method.

‡Test for difference in imputation accuracy proportions across individuals with 0, 1, or 2 West African alleles at each gene.

Finally, to determine the benefit of adding more genetic variants via imputation prior to model construction and during HIBAG imputation, we compared ACCESS HIBAG results (Table 2) against two different imputation strategies: ACCESS observed plus 1KG-imputed data; and ACCESS observed plus 1KG and targeted sequencing-imputed data (Table 6). These models performed well (imputation accuracy > 90%) at two-field resolution across all genes. When the suggested posterior probability threshold of 0.5 was applied, we observed better call rates while maintaining high imputation accuracies when imputing more variants for *HLA–DRB1* (6.1%–21.6% improved), *–DQB1* (1.1%–10.6% improved), and *–DPB1* (1.8%–5.4% improved). These results highlight the contribution of increased SNP density to overall imputation quality and accuracy. Because the sequencing region captured only *HLA–DRB1* and *–DQB1*, we were not able to test the accuracy of a model using both the 1KG and targeted sequencing reference panels for *HLA–DPB1*.

**Table 6:** **The effect of adding imputed SNPs to the HIBAG *HLA *** **allele imputation accuracy in ACCESS African Americans for *HLA *** **class II genes**

	**Gene**	**Allele field**	**Testing set**				
			**All**		**PP > 0.5**		
			**N**	**Accuracy**	**N**	**Call rate**	**Accuracy**
ACCESS Subset* - GWAS + 1000GP Imputed data^a^	*DRB1*	One	164	95.4	149	90.9	97.0
		Two	164	90.2	123	75.0	95.9
	*DQB1*	One	188	99.2	185	98.4	99.5
		Two	188	98.1	168	89.4	99.4
	*DPB1*†	One	250	87.3	160	64.2	97.5
		Two	250	91.8	151	60.6	97.0
ACCESS Subset* - GWAS + 1000GP + TargetSeq Imputed data^b^	*DRB1*	One	164	94.8	147	89.6	97.0
		Two	164	89.6	129	78.7	95.0
	*DQB1*	One	188	99.2	186	98.9	99.2
		Two	188	97.9	171	91.0	99.4

Abbreviations: PP denotes, posterior prediction probability; N, count; Accuracy, imputation accuracy.

*The models trained on the subset of ACCESS African Americans genotype data from ^a)^GWAS + 1000 Genome project imputed data, ^b)^GWAS + 1000 Genome project + targeted sequencing imputed data (*DRB1* and *DQB1* only) with training samples selected at random (from a total of 325) using to match the number of subjects used to construct the published HIBAG African ancestry models: *HLA-DRB1* (n=161), *HLA-DQB1* (n=137), and *HLA-DPB1* (n=75).

†One- and two-field estimates will be very similar for *HLA-DPB1* as the first field uniquely identifies the two-field alleles, with the exceptions of *02:01 and *0202, and *04:01 and *04:02.

## Discussion

Data on *HLA* alleles are essential for understanding causal variation that underlies SNP associations found in GWAS of diseases with a strong *HLA* component. Despite reductions in the cost of genome-wide genotyping in GWAS, genotyping *HLA* alleles remains expensive, although less expensive next-generation sequencing methods now exist [26,27]. The cost of *HLA* allele typing in large samples has spurred the development of methods using data on common variants from GWAS genotyping, as many existing studies already possess this data. Such methods can be a low or no-cost option for studies with existing data.

Our results for European Americans validate the high one- and two-field accuracy rates reported for the validated HLA*IMP:02 method [7,8]. In our sample, the HIBAG results are similar or better. HIBAG also performed well in an African American sample; reductions in overall imputation accuracies compared to those from European American samples are partially due to sample sizes in the training set. When two-field allele sensitivity estimates are compared by allele frequency, our findings suggest that rare alleles are more susceptible to poor imputation, even given large reference panels. These findings agree with those of Leslie et al. [7] that showed the sensitivity of two-field allele prediction was related to occurrences of the allele in the model training data. Further, in smaller reference panels—such as those currently available for admixed populations—even relatively common alleles may be poorly imputed. While these findings suggest that *HLA* allele imputation accuracy in admixed populations could benefit from increasing the number of reference haplotypes, our results also suggest additional causes of low imputation accuracy in African Americans.

When we used equivalent samples sizes and compared models constructed on the sample of African Americans from ACCESS to those from published HIBAG African ancestry models, we found consistent increases in imputation quality and accuracy from the ACCESS models. One possible reason is SNP density in the models. The HIBAG models were constructed using a subset of SNPs overlapping across three different Illumina GWAS platforms [16], whereas our models included all the SNPs from only one platform. The improvement in accuracy related to the density of SNPs and completeness of genotyping (*i.e.* decreasing levels of missing calls for genotyped SNPs following imputation) is also supported by our results from different imputation strategies.

Another source of improvement may be related to complexity in the ancestral origins of individuals included in the training samples. For example, the training samples included in published HIBAG African ancestry models may encompass sub-populations that differ from ACCESS African Americans in their *HLA* allele frequency spectrum. In addition to African Americans and Yoruba individuals who were part of the International HapMap project and originally genotyped for the *HLA* alleles by de Bakker et al. [6], the HIBAG African ancestry sample included individuals from South Africa. This population is not thought to have contributed substantively to the genomes of present day African Americans and may not be informative for *HLA* imputation in this population. Further, our findings suggest that consideration of local ancestry can aid in the improvement of *HLA* allele imputation accuracy in admixed populations, as training-set results for individuals heterozygous for local West African ancestry were inferior to those for homozygous individuals. An informed sampling of ancestral haplotypes may be necessary to produce high-quality predictions in the admixed population of interest.

Based on our results that suggest denser SNP genotyping may be related to improved imputation accuracy, these findings suggest that accuracy could be improved in admixed populations through direct *HLA* allele genotyping in a large, geographically-diverse reference sample of individuals with complete sequencing of the broader *MHC* region, such as the 1KP [28]. Use of the near-complete catalogue of SNPs in the model building process would eliminate the need to account for genotyping platform.

Genetic association studies are one of the principal applications of GWAS SNP-based *HLA* imputation; in this setting, accuracy and quality of imputation is directly related to power. When applying a posterior probability threshold of 0.5, we found that imputing more variants in the training set improved call rates (and thus the sample size) while maintaining high imputation accuracy. The improvements in call rate were most dramatic for the strategy that included targeted sequencing for *HLA–DRB1* and *–DQB1*, which is likely the result of direct genotyping and subsequent imputation of the non-synonymous polymorphisms that define the *HLA* alleles at two-field resolutions. While the gains in call rate may seem negligible, when prediction is used to test the association of these alleles with a trait of interest, even modest increases in sample size may dramatically impact statistical power.

For applications other than genetic association mapping, additional metrics may be more appropriate. Clinical pharmacogenetic applications, such as the identification of patients likely to experience *HLA*-associated adverse drug reactions (*e.g.* abacavir hypersensitivity in carriers of HLA-B*57:01 [29]), may benefit from a metric that can account for uncertainty in predictions as well as differentiate between correct and incorrect classification. In such cases, the generalized Bayesian information reward applied to machine learning classification methods [30] may be a solution. This method compares the natural logarithm likelihood of the model (based on posterior probability of the observed genotypes estimated from HIBAG) to a null model (expected genotype frequencies in the population, assuming Hardy-Weinberg Equilibrium). For our purposes, however, imputation accuracy is a valid method for evaluating the relative strengths and weaknesses of different imputation modeling strategies.

*HLA* allele prediction in non-European populations has not been widely reported on in the literature, likely due to lack of methods and references panels. Recently published allele prediction results for *HLA–DRB1* and *–DQB1* in the Wolita population of southern Ethiopia [31] used a multi-allelic prediction model [11] that was accurate at the one-field level (>85%) but less so at the two-field level (<32%). These models were constructed using just 10 and 19 SNPs for *–DRB1* and *–DQB1* respectively, which may explain the low two-field accuracy [32]. Such results demonstrate the need for larger reference panels of *HLA* alleles and dense SNP genotyping in the *HLA* region for non-European populations.

The HIBAG approach has several practical advantages over other established *HLA* imputation methods. First, researchers can build models for prediction using their own samples, particularly in non-European populations for whom a reference panel has not been established. In African Americans, ancestral contributions are primarily of West African and European origin; admixed populations with greater ancestral heterogeneity (*e.g.* Latinos [33-38]) likely require additional population-specific reference panels to improve imputation accuracy. Further, HIBAG uses the open-source R statistical programming language. Models produced by researchers can be shared without proprietary software or the transfer of protected health information such as individual genotype data. The African American *HLA* class II imputation models produced for this study are available on request.

A limitation of this study is our use of *HLA* typing data that is over a decade old as our gold standard to estimate imputation accuracy. We recognize these data are incomplete in terms of the current compendium of *HLA* class II alleles, but it should be noted that even today’s high-resolution *HLA* typing results in ambiguous allele and genotype calls (*i.e.* multiple distinct alleles consistent with the same raw sequence). While the undetected or misclassified alleles in our *HLA* typing are not strictly quantifiable, our estimates of imputation accuracy should be conservative, since *HLA* typing misclassification will likely decrease our estimate of imputation accuracy. Despite the limitations of our *HLA* typing data, our findings are similar to the ethnicity-specific accuracy estimates reported in the HIBAG [16] and HLA*IMP:02 [13] manuscripts that both used allele typing based on more recent versions if the IMGT/HLA Database. Due to recent gains in knowledge regarding the differences in *HLA* allele frequencies worldwide [39-42], larger representative reference panels coupled with current *HLA* allele typing should lead to improvements in imputation of lower frequency alleles in admixed populations such as African Americans.

## Conclusions

In conclusion, our study suggests that the newly developed HIBAG approach is appropriate for use of *HLA* class II imputation in both European and admixed non-European populations. Imputation quality is closely associated with *HLA* allele frequency, training sample size, SNP density, and how well the training sample represents the test sample in ancestral origin. The latter point is especially true for admixed populations, where our findings suggest that accounting for local ancestry in the selection of the training samples will be beneficial. Additionally, applying next-generation targeted sequencing data, when available, may boost both HIBAG imputation accuracy and certainty in modest samples sizes of admixed individuals. We expect that these results are generalizable to other African admixed populations and should be useful in any study seeking to better characterize the role of *HLA* class II alleles.

## Methods

### Consent and ethics statement

As stated above, data for this study was derived from four prior studies [22-25]. For each of these studies, participants gave written informed consent to allow their research material to be used in future genetic studies. Study protocols were approved by the institutional review board of each study site (Beth Israel Deaconess Medical Center, Boston, MA; Cleveland Clinic, Cleveland, OH; Emory Healthcare, Atlanta, GA; Georgetown University Medical System, Washington, DC; HFHS, Detroit, MI; Johns Hopkins Hospital, Baltimore, MD; Medical University of South Carolina, Charleston, SC; Mount Sinai Hospital, New York, NY; National Jewish Hospital, Denver, CO; University of Cincinnati Hospital, Cincinnati, OH; University of Iowa Health Care, Iowa City, IA; University of North Carolina Medical Center, Chapel Hill, NC; University of Pennsylvania Health System, Philadelphia, PA).

### Genotyping

Details of the molecular allele typing are reported in Rossman et al. [18]. Briefly, *HLA* typing was conducted over exon 2 for the class II genes *–DRB1*, −*DQB1*, and *–DPB1*. Low (one-field) to intermediate (two-field) resolution typing was performed with sequence-specific oligonucleotide probes available through Orchid Diagnostics [18], in the context of the version 1.13 release of the IMGT/HLA 2002 database. Genotyping was performed using the Illumina HumanOmni1-Quad [17]. SNPs meeting the following quality control criteria were included: well-defined cluster plots by visual inspection; call rate greater than 90%; minor allele frequencies greater than 0.001; and p-value greater than 0.001 for Hardy-Weinberg proportion tests in controls.

### Targeted resequencing, variant detection, and quality control

Purified genomic DNA from 480 African American individuals (187 sarcoidosis cases, 293 controls) was prepared for sequencing using the Illumina Paired-End DNA Sample Preparation Kit (San Diego, CA). The Illumina TruSeq technology with a custom-designed bait pool was used to enrich captured regions (including *HLA–DRB1* and *–DQB1*). Resequencing and generation of fastq sequencing reads were performed on the Illumina HiSeq2000 platform with Illumina Pipeline software (version 1.7). After removing duplicates, alignment to the Human Reference Genome (build hg19) was performed with BWA alignment software (version 0.5.9) [43]. Realignment around insertion/deletion sites, base quality score recalibration, and variation detection were carried out using GATK software (version 1.0) [44,45]. Variants displaying any of the following were removed: quality score <30; by-depth score <5; strand bias score > ^−^0.1; homopolymer runs ≥5 bases; or variants clustering within 10 base pairs. Average sequence coverage was 75x. Three samples were removed because of low genotype call rates (<95%). Variant phasing was performed using BEAGLE (version 3.3) [12]; PLINK (version 1.07) [46]; and IMPUTE2 [47]. File formatting was performed with VCFtools (version 0.1.3) [48]. IMPUTE2 [47] was used to impute variants spanning chromosome 6p21, with targeted sequencing data and the 1KG Phase I integrated variant set as reference panels. Variants with low imputation accuracy (information measure <0.5; average maximum posterior genotype call probability <0.9) or failing to meet quality-control standards (described above) were excluded. Imputation was performed using the 1KG data over a region on chromosome 6 starting at 31,842,535 bp and extending to 33,720,220 bp, which included *HLA–DRB1*, −*DQB1*, and *–DPB1*; targeted sequencing data was available for the region starting at 31,842,535 bp and extending to 32,720,220 bp, which included *HLA–DRB1* and *–DQB1* only.

### Statistical analysis

For European American subjects, HIBAG [16] was compared to the HLA*IMP:02 [7,8,13], based on the latest version of each method with a reference population appropriate for African Americans. Predictions for African Americans were also compared with those from BEAGLE [12], which allows for simultaneous haplotype phasing and imputation of genetic markers with two or more alleles.

As part of the initial HIBAG method development, ancestry-specific prediction models were evaluated in independent discovery and validation samples from individuals of primarily European, African, east Asian, and Latino ancestry for *HLA–A*, −*B*, −*C* , −*DPB1*, −*DQA1*, −*DQB1*, and *–DRB1* genes. We used these existing models to evaluate imputation accuracy in the subset of ACCESS African Americans and European Americans with both GWAS and *HLA* allele typing available for –*DPB1*, −*DQB1*, and –*DRB1*. Imputation accuracy was based on the overall classification rate for each ethnicity/gene combination, as well as for the individual alleles at both one- and two-field resolution. It should be noted that the one- and two-field resolution estimates will be very similar for *HLA-DPB1* as the first field uniquely identifies the two-field alleles with the exceptions of *02:01 and *02:02, and *04:01 and *04:02 [49]. These estimates were determined first on all HIBAG calls, regardless of the posterior probability of the genotype prediction, and then on the subset of predictions with >50% posterior probability. Many additional alleles have been added to the IMGT/HLA database in the years since the *HLA* data was generated by Rossman et al. [18], and some of these are identical over exon 2 with the alleles from the original genotyping. These ambiguous alleles are catalogued as part of the IMGT/HLA database; we used information from the 2014 version (3.15.0) to resolve predicted allele differences between the prebuilt HIBAG and HLA*IMP:02 models in comparison with our *HLA* genotype data.

Models were also constructed from random samples of African American individuals from the ACCESS sample; sample sizes were selected to match each of the gene-specific published HIBAG African ancestry models (n = 161 for *HLA–DRB1*; n = 137 for *HLA–DQB1*; n = 75 for *HLA–DPB1*). Accuracy estimates from published HIBAG models were compared to those from samples not included in the model training process. These ACCESS African American models were constructed using genotype data from variants located 500 kb away from the largest mRNA transcript for each gene in the NCBI RefSeq database, resulting in 3,144 (*HLA–DRB1*), 3,417 *(−DQB1*), and 2,473 (−*DPB1*) markers. For the ACCESS HIBAG models, we report the SNPs used in each HIBAG model in Additional file 5 and Additional file 6, and as multiple classifiers are included in each model, the count of the number of classifiers each SNP appeared in is also included. Imputation based on 1KG and targeted sequencing data added an additional 10,935 variants for *HLA-DRB1* (9,950 1KG; 985 targeted sequencing), 11,376 variants for *HLA-DQB1* (10,475 1KG; 901 targeted sequencing), and 8,889 for *HLA-DPB1* (8,889 1KG).

To evaluate the effect of local West African ancestry on imputation accuracy, we estimated local ancestry (0, 1, or 2 alleles of West African origin) at each GWAS marker across the *MHC* locus using the Local Ancestry in adMixed Populations method [20,50]. Ancestral European and West African allele frequencies were obtained from HapMap catalogs [51] made available through Illumina iControl. For each class II gene, classification accuracy was estimated for each local ancestry state, and a Fisher’s exact test was used to test for differences in classification accuracy (assuming type-1 error of 0.05).

## Abbreviations

HLA denotes: Human leukocyte antigen; SNP: Single nucleotide polymorphism; HIBAG: HLA genotype imputation with attribute bagging; MHC: Major histocompatibility complex; GWAS: Genome-wide association studies; 1KG: 1000 genomes project; ACCESS: A case–control etiologic study of sarcoidosis.

## Competing interests

The authors declare that there are no competing interests associated with this work.

## Authors’ contributions

AML conceived of the study, participated in the design of the study, performed statistical analyses, and drafted the manuscript. IA carried out the genome-wide genotyping, targeted sequencing, quality control, performed statistical analysis, and drafted the manuscript. ID performed statistical analysis and aided in the drafting of the manuscript. MC, ST, and PM participated in the design of the study and drafting the manuscript. CGM and BAR conceived of the study, participated in its design and coordination, and helped to draft the manuscript. All authors read and approved the final manuscript.

## Authors’ information

CGM and BAR share senior authorship of this paper.

## Supplementary Material

Additional file 1 “HIBAG European Ancesty allele prediction measures for ACCESS European Americans”. Click here for file

Additional file 2 “HIBAG African Ancesty allele prediction measures for ACCESS African Americans”. Click here for file

Additional file 3 “ACCESS African American HIBAG model allele prediction measures for ACCESS African Americans”. Click here for file

Additional file 4 “Allele prediction measures from ACCESS HIBAG models composed of equal numbers (n = 150) of ancestral West African and European chromosomes”. Click here for file

Additional file 5 **“The SNPs HIBAG utilized in the ACCESS gene-specific models with results displayed in Table **2**”.** Click here for file

Additional file 6 **“The SNPs HIBAG utilized in the gene-specific ACCESS local ancestry-balanced models with results displayed in Table **5**”.** Click here for file
